# Does Relative Energy Deficiency in Sport (REDs) Syndrome Exist?

**DOI:** 10.1007/s40279-024-02108-y

**Published:** 2024-09-17

**Authors:** Asker E. Jeukendrup, Jose L. Areta, Lara Van Genechten, Carl Langan-Evans, Charles R. Pedlar, Gil Rodas, Craig Sale, Neil P. Walsh

**Affiliations:** 1 Loughborough University, Loughborough, UK; 2 Netherlands Olympic Committee, Arnhem, The Netherlands; 3 Liverpool John Moores University, Liverpool, UK; 4 St Mary’s University, Twickenham, London, UK; 5 Medical Department, Futbol Club Barcelona, Barça Innovation Hub, Barcelona, Spain; 6 Manchester Metropolitan University, Manchester, UK

## Abstract

Relative energy deficiency in sport (REDs) is a widely adopted model, originally proposed by an International Olympic Committee (IOC) expert panel in 2014 and recently updated in an IOC 2023 consensus statement. The model describes how low energy availability (LEA) causes a wide range of deleterious health and performance outcomes in athletes. With increasing frequency, sports practitioners are diagnosing athletes with “REDs,” or “REDs syndrome,” based largely upon symptom presentation. The purpose of this review is not to “debunk” REDs but to challenge dogmas and encourage rigorous scientific processes. We critically discuss the REDs concept and existing empirical evidence available to support the model. The consensus (IOC 2023) is that energy availability, which is at the core of REDs syndrome, is impossible to measure accurately enough in the field, and therefore, the only way to diagnose an athlete with REDs appears to be by studying symptom presentation and risk factors. However, the symptoms are rather generic, and the causes likely multifactorial. Here we discuss that (1) it is very difficult to isolate the effects of LEA from other potential causes of the same symptoms (in the laboratory but even more so in the field); (2) the model is grounded in the idea that one factor causes symptoms rather than a combination of factors adding up to the etiology. For example, the model does not allow for high allostatic load (psychophysiological “wear and tear”) to explain the symptoms; (3) the REDs diagnosis is by definition biased because one is trying to prove that the correct diagnosis is REDs, by excluding other potential causes (referred to as differential diagnosis, although a differential diagnosis is supposed to find the cause, not demonstrate that it is a pre-determined cause); (4) observational/cross-sectional studies have typically been short duration (< 7 days) and do not address the long term “problematic LEA,” as described in the IOC 2023 consensus statement; and (5) the evidence is not as convincing as it is sometimes believed to be (i.e., many practitioners believe REDs is well established). Very few studies can demonstrate causality between LEA and symptoms, most studies demonstrate associations and there is a worrying number of (narrative) reviews on the topic, relative to original research. Here we suggest that the athlete is best served by an unbiased approach that places health at the center, leaving open all possible explanations for the presented symptoms. Practitioners could use a checklist that addresses eight categories of potential causes and involve the relevant experts if and when needed. The Athlete Health and Readiness Checklist (AHaRC) we introduce here simply consists of tools that have already been developed by various expert/consensus statements to monitor and troubleshoot aspects of athlete health and performance issues. Isolating the purported effects of LEA from the myriad of other potential causes of REDs symptoms is experimentally challenging. This renders the REDs model somewhat immune to falsification and we may never definitively answer the question, “does REDs syndrome exist?” From a practical point of view, it is not necessary to isolate LEA as a cause because all potential areas of health and performance improvement should be identified and tackled.

## Key Summary Points

**Table:** 

Relative energy deficiency in sport (REDs) is a widely adopted model describing how low energy availability (LEA) causes a wide range of deleterious health and performance outcomes in athletes.
Empirical evidence supporting the REDs model is limited.
REDs symptoms may be caused by many factors, independent of or co-occurring with LEA, including poor mental health, disordered eating and eating disorders, poor sleep, infection, injury and undiagnosed clinical conditions.
The Athlete Health and Readiness Checklist (AHaRC) is presented as a multidimensional monitoring tool to identify the likely cause(s) of symptoms and ensure that practitioners select appropriate guidance and treatment, where necessary.

## Introduction

Relative energy deficiency in sport (REDs) is a model that built upon the female athlete triad work first presented in 1993 [1] with a female athlete triad consensus paper published in 1997 [2]. REDs was first introduced in 2014 [3] and describes how inadequate energy intake, for the demands of training [i.e., low energy availability (LEA)] by athletes, “causes” a wide range of symptoms far beyond the health symptoms discussed in the female athlete triad, including effects on performance [4]. This wide range of symptoms include amongst others menstrual irregularities, poor bone health, compromised immune function, reductions in performance, fatigue and poor mental health.

The number of publications on the topic of REDs has risen significantly since the concept was first introduced [3]. Although REDs was originally described as a model [3], in sports practice, athletes are now being diagnosed with “REDs” or “REDs syndrome” [4, 5] and it may appear that REDs is well-established. We even see dedicated REDs clinics (examples [6–8]). REDs has become a much-discussed topic on social media and in mainstream media news outlets (examples: [9–11]). However, as the REDs concept is nascent, the mediatic growth is incommensurate with the scientific evidence, and the number of clinical trials showing a causal effect of LEA is limited.

The first consensus statement by the IOC on this topic [3] encouraged readers to never stop questioning. Herein, we have taken up this challenge and have undertaken an examination of the REDs model. Much of the support provided for the model is from studies that have used simple questionnaires, or poor measurements of energy availability, whereas the current consensus [4] clearly indicates the difficulties measuring energy availability as well as limitations using questionnaires without measurements of bone mineral density (BMD) and a series of other biomarkers (as evidenced by the new REDs CAT-2 tool). Many researchers and practitioners are working on the basis that REDs is a well-established phenomenon and that LEA and REDs are highly prevalent in athletes [12–16] with studies even reporting 100% of athletes with LEA [17, 18] and up to 80% being at risk or suffering from REDs [12, 16]. We believe this is possibly misleading and may prevent the researcher and practitioner communities from unveiling the multifactorial causes of common symptoms observed in athletes.

**Fig. 1:**
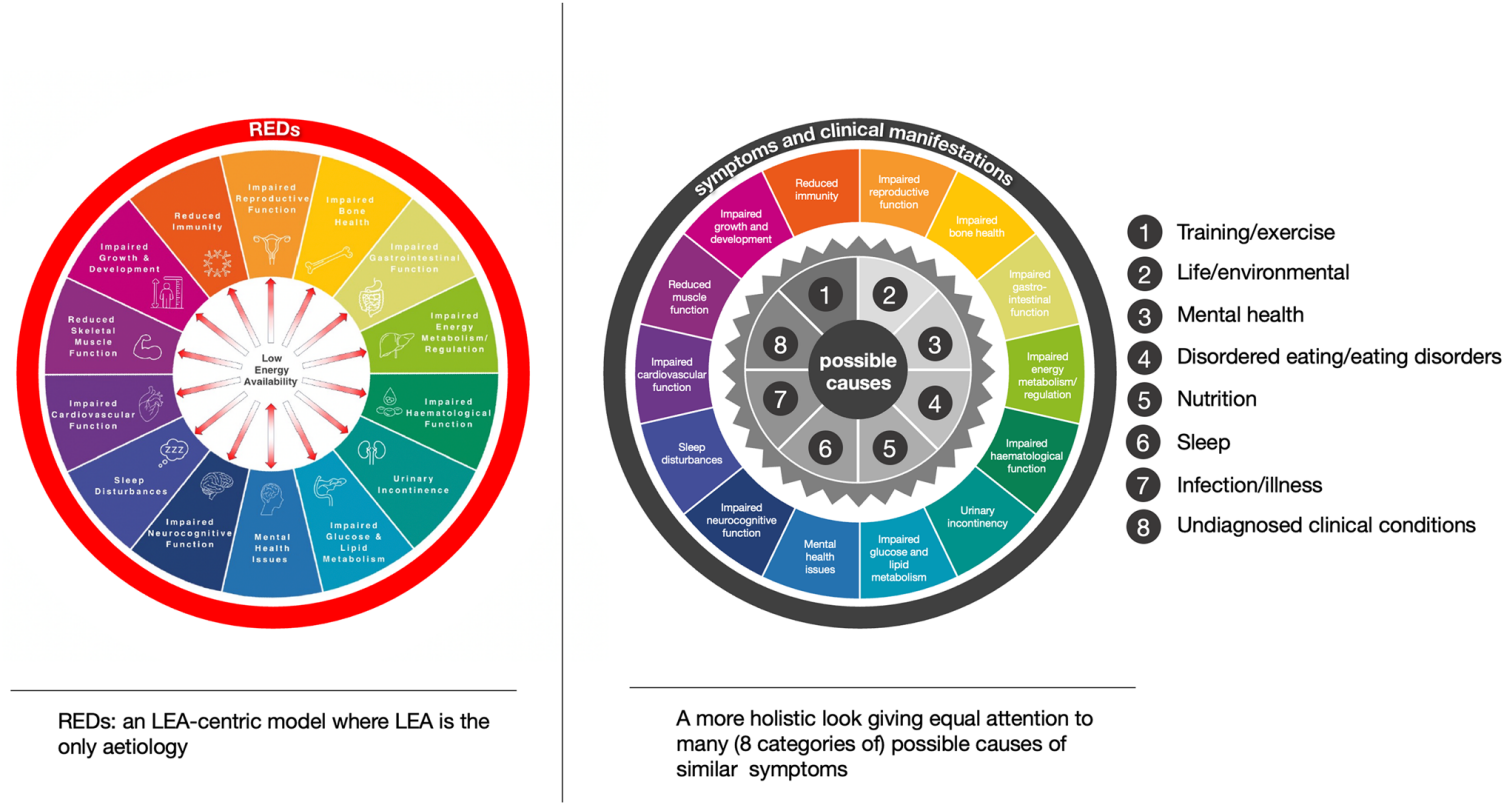
Comparison of the current REDs model that centers around LEA as the only cause of symptoms (Left: with permission [4]) and the more holistic approach we are presenting in this paper

The main goal of this article is to provide a critical review of the concept of REDs to help advance the science, have an objective view on the strength of the available evidence behind the concept (focused on the most researched aspects of REDs), and ask the pragmatic question, “what is the best way to support the health and performance of athletes?” Herein we suggest that the REDs model is too “calorie centric” and propose a more holistic and comprehensive approach in which LEA is one of many potential causes of the symptoms described by the model (Fig. 1).

Instead of LEA being the single cause of REDs symptoms we propose that there are many causes (in eight categories). These causes can act independently or in combination and LEA can be one of these many causes (or moderators). We are not proposing a new model, rather we are stating that models developed in the 1950s and 1990s (the general adaptation syndrome and the allostatic load model) are more comprehensive and are more suitable to explain the REDs symptoms in athletes (Sect. 3). This is not the first time the REDs model has been evaluated and critiqued. For example, in one critical paper [19] the authors question several assumptions and methods and ask for more research. Several years later attempts have been made to address some but not all of these questions. In the meantime, the REDs model has also evolved attempting to address some of the concerns, but this has also raised new questions. For example, the latest consensus paper stresses that diagnosis of REDs cannot be performed directly and would need to be made through measurements of symptoms and exclusion of other causes. The model has evolved into including even more symptoms and even more potentially affected body systems without providing robust evidence to support such claims. Most importantly, in this process of diagnosing by exclusion, the assumption, and a bias, is that symptoms are directly caused by LEA, unless there is another cause. We will introduce an Athlete Health and Readiness Checklist (AHaRC), which has a number of tools from other expert/consensus statements, that together will provide a more holistic and less biased approach to athlete health.

### Historical Perspective

In 1993 the female athlete triad was proposed by authors researching eating disorders, menstrual function and bone mineral density (BMD) in athletes [1]. A common co-existence of the clinical manifestations, amenorrhea and osteoporosis, was reported in individuals with eating disorders. In 1997 a triad consensus statement was published referring to LEA as a hypothetical factor causing amenorrhea and osteoporosis in the presence or absence of an eating disorder [2]. At this point in time there was little experimental work to support the claims, but seminal research by Loucks and colleagues, aiming to separate the effects of energy availability (EA) and exercise in a series of well-designed studies, established causal links between LEA and alterations of the endocrine milieu and markers of bone metabolism and BMD [20–23]. A 2007 female athlete triad consensus statement made a major modification replacing “eating disorders” with “low energy availability” [24]. In 2014, a new model, the RED-S (now *REDs*), was proposed to build upon and expand the female athlete triad model [3].

This REDs model suggested that LEA could have several health effects in addition to amenorrhea and osteoporosis including, but not limited to, effects on immune function, gastrointestinal function, and cardiovascular function [3]. It was also stated that male athletes were at a lower risk for developing eating disorders [25, 26], but there were links between LEA and BMD in males [27]. In contrast to the female athlete triad, where the diagnoses were eating disorders, amenorrhea, or osteoporosis, REDs was now also presented as a diagnosis, a syndrome with many potential symptoms. It was acknowledged that the screening and diagnosis of REDs was challenging [3]. This statement was refined 4 years later in a new consensus statement by the IOC [28]. Some of the changes included a more comprehensive discussion of the effects in male athletes, a discussion of the complications of assessing LEA, and it was stated that one of ultimate goals was to stimulate awareness of the effects of LEA [28].

The definition of REDs has evolved over time, but in the latest IOC consensus statement it is defined as a syndrome of impaired physiological and/or psychological functioning experienced by female and male athletes that is caused by exposure to problematic (prolonged and/or severe) LEA [4]. The detrimental outcomes include, but are not limited to, decreases in metabolic function, reproductive function, musculoskeletal health, immunity, glycogen synthesis, and cardiovascular and hematological health, which can all individually and synergistically lead to impaired well-being, increased injury risk, and decreased sports performance [4].

The most recent REDs IOC consensus statement attempted to address some of the shortcomings of the previous statements, and reporting on advancements in the field [4]. Some of the main changes included: an expanded range of symptoms, recognition that not all LEA has negative health or performance outcomes, and a wide array of differential diagnoses. Some LEA was classed as “adaptable,” with REDs specifically being the outcome of “problematic” LEA [4]. The consensus does not, however, cite any primary evidence or objective parameters to provide clarity on how adaptable and problematic LEA could be separated, other than waiting for symptoms to develop. This is a limitation because most studies used to support the REDs model have studied adaptable LEA and not problematic LEA. The new consensus recognizes that other factors may play a role in the etiology of REDs and can even be independent of EA, but these are referred to as “moderating factors” [4]. Importantly, a supporting paper provides an extensive list of moderating factors that need to be considered, including many different etiologies (differential diagnoses) that could generate the signs or symptoms of REDs [29]. The list of factors to be considered and excluded, is likely far from complete and is not practical for practitioners or clinicians. Perhaps this was beyond the scope of the consensus, but it is central to the task of helping athletes to maintain health and performance.

Finally, it is worth highlighting a major logical fallacy in the fundamental tenet of the REDs model. As it is stated: “REDs (by definition) is a collection of symptoms that are caused only by problematic LEA” [4]. It is only possible to diagnose problematic LEA through the measurements of symptoms. But then the same statement acknowledges “there are other causes of these symptoms” [4]. Therefore, it appears that a calorie-centric (energy-centric) model may be a paradigm that predisposes the observer to bias, inflicting at least partial blinding of several other common etiological factors that may be exerting responses attributed only to LEA; a more holistic approach is warranted.

### The Definition and Calculation of Energy Availability

Previous visualizations of the REDs model placed REDs as the hub of a wheel with a large number of spokes [3], representing categories of symptoms, although this seems to have been in error, since REDs refers to the symptoms and not the cause, which has always been said to be LEA. This has been corrected in the most recent consensus [4] in which LEA is displayed as the only cause of a wide range of symptoms and the overall syndrome is referred to as REDs. The current definition of EA refers to the dietary energy available to sustain normal physiological function after exercise energy expenditure is subtracted, and in its latest form the algebraic definition is represented as [30]:

$${\text{EA}} = \left( {{\text{energy}}\;{\text{intake}} - {\text{net}}\;{\text{exercise}}\;{\text{energy}}\;{\text{expenditure}}} \right)/{\text{fat free mass}}\;({\text{FFM}})$$

It is generally considered that an EA of ~ 45 kcal^.^kg FFM^−1^.day^−1^ is healthy, but an EA below 30 kcal^.^kg FFM^−1.^day^−1^ is problematic for health. These levels have been largely based upon two instrumental studies that titrated EA levels in habitually sedentary women who performed exercise to 10, 20, 30, and 45 kcal^.^kg FFM^−1.^day^−1^ for 5 days; showing that with EA at or below 30 kcal^.^kg FFM^−1.^day^−1^ alterations in hormonal and metabolic markers occurred [21, 22, 31]. These changes were consistent with alteration of the hypothalamic pituitary gonadal (HPG) axis hormones that are mechanistically linked to the cause of amenorrhea [21, 22, 31] and changes in markers of bone resorption and formation, with a reduction in bone formation indicated in each LEA condition and an increase in bone resorption indicated at more severe LEA [32].

Although this mathematical equation of establishing EA seems very straightforward, the reality is that the calculation lacks accuracy [33] for several reasons:

- Interpretation of what net energy expenditure entails is not uniformly agreed or applied i.e., there is no consensus on what is classified as “exercise” and what is “non-exercise activity thermogenesis.”
- Assessment of energy intake is usually done using food diaries which are known to result in underreporting with errors of up to 60% (19% on average [34]).
- The accuracy of estimations of energy expended during exercise will vary greatly by sport, and will be dependent upon factors such as the device used (often accelerometers, or heart rate monitors), whether or not activity is self-reported, mechanical efficiency, and so on [35].

Measurements of FFM are typically achieved through generic equations. These equations vary significantly, may be established in different populations, and will only provide an approximation of an individual’s FFM. Errors upwards of 2–3% [36] are likely with gold standard methods (and standardization of measurement procedures), but at an individual level and with techniques often used in sports practice (such as bioelectrical impedance and skinfolds) these errors can be significantly larger due to accumulation of measurement error and day to day variation.

It is now generally agreed that, in practical settings, it is questionable whether estimations of LEA can be obtained that are reliable enough to base sound conclusions upon [4, 33]. In addition, such measurements only provide a snapshot and would not necessarily be representative of the preceding weeks, months or years. Finally, a fixed cut off value for EA of 30 kcal^.^kg FFM^−1^ day^−1^ does not consider the possible inter-individual variation in responses to EA [37]. Especially in real-life settings when the direct assessment of LEA cannot be relied upon for the diagnosis of REDs [4, 33]. The use of cut-off values was abandoned in the last IOC consensus paper [4, 33]. This does not solve the issue: how would we ever know if someone is in LEA if the measurements are not accurate and we do not have cut-offs?

It is also noteworthy that even though field-based EA assessment is fraught with error, a significant number of observational studies adopt field-based EA assessment [38–43]. Many of these studies interpret outcomes of these EA assessments as accurate, with little or no consideration for the error of measurement. In many cases this has resulted in classification of athletes being in LEA (“clinical” or “subclinical”). It is more than likely that this has resulted in an over-estimation of the prevalence and severity of LEA. In a recent study in female football players the average EA was 34 ± 12 kcal^.^kg FFM^−1.^day^−1^ with six players (34%) reported to meet the criteria for LEA (below the threshold of 30 kcal^.^kg FFM^−1.^day^−1^). When corrected for potential underreporting (based on [44]), however, the average EA was 44 kcal^.^kg FFM^−1.^day^−1^ with only one player (5%) meeting the criteria for LEA) [45]. Other studies have been more careful and more critical in their interpretation of the data [46, 47] but still use the same methods or the cutoffs that have been abandoned and a single short duration assessment period.

### Why Diagnosing REDs by Symptom Presentation Alone is Flawed

As it is not possible to use the mathematical model and calculate LEA accurately in practice, and because even with such a calculation it is not feasible to distinguish adaptable from problematic LEA, it has been proposed that the only approach that will work is to measure the outcome (symptoms) [33, 48]. Several questionnaire-based tools have been developed to estimate the risk of LEA, including the LEAF-Q [49], LEAM-Q [50], and RED-S-CAT [51] (succeeded by REDs-CAT2 [52]). A recent review counted eight questionnaires that claimed to be validated plus three more questionnaires, all with the aim to predict LEA [53]. There is clearly no uniform way of assessing symptoms and these tools have no inherent consideration for the fact that the symptoms could be caused by other factors that are either related to, or completely independent of LEA. The tools are usually validated in one specific population but are sometimes applied across a wide range of athlete populations.

There is a flaw in the reasoning that measuring common symptoms can provide information about the cause, unless all other important factors are considered. An analogy would be that if we have studies showing that excess energy intake in the form of sugar can result in weight gain, and there are studies showing that weight gain can result in obesity over time, and obesity is associated with various negative health outcomes, then, if we measure these health outcomes in an individual, can we conclude that the person ate excessive amounts of sugar? Of course, this conclusion would be flawed because there are many other factors that need to be considered in the development of obesity and related health consequences. Some of these factors could be nutrition related (for example, fat or alcohol intake), but also social factors, psychological factors and many environmental factors like infrastructure would need to be considered. We would not call all these factors “moderators” of the effect of excessive sugar intake. They are just other causes that also need to be considered.

### REDs is a Model, and a Model is a Simplified Representation of Reality

Models are useful for making a particular part or feature of the world easier to understand, define, quantify, visualize or simulate. A model is an idealized (simplified) representation of aspects of the world around us, simplifying physical, biological or sociological phenomena.

The model does this by referencing existing and usually commonly accepted knowledge. Although models are indispensable for biomedical research they need to be tested, and in this process, it is usually discovered that the model is inadequate or even incorrect and needs to be adjusted or replaced by a different model. REDs is a model [3] and like any other model it needs to be scrutinized and improved. The group that was responsible for the consensus statements will continue to do this, but others should also scrutinize the ideas, the assumptions and theories. In mainstream media, however, the idea is presented as a fact and the large number of narrative reviews and consensus statements suggest that it is well-established. We propose that the model could be adapted or a new model could be developed with less bias towards insufficient calorie intake as the only cause. The approach should be more open-minded with the goal of finding the cause, rather than trying to prove that LEA is the cause. There are other equally likely, or sometimes more likely, causes of the symptomology that need to be considered.

In the following sections, we discuss the underlying evidence for the REDs model highlighting that it has not been as thoroughly tested as is often claimed and therefore remains a model that may have to be adjusted or maybe even abandoned or replaced.

## How Strong is the Evidence?

In the last few years there has been a significant increase in the number of publications in this area. A search on PubMed using the terms “energy availability” AND “exercise” OR “athlete” (20 Sept 2023) provided 440 papers. Approximately 40% were narrative reviews, commentaries and editorials and 60% were empirical research studies, of which roughly 90% were observational and less than 10% RCTs (i.e., able to show a causal relationship between LEA and study outcomes). Much of the empirical research quoted consists of older studies that were used as support for the female athlete triad model, indicating that the area has advanced little since this original work. It is also important to note that most of the empirical research studies (70%) were short term (less than 7 days in duration), and only 29% of the studies investigated athletes, and even fewer elite athletes. Importantly, there was no evidence of experimental studies to support the majority of the proposed health and performance consequences of REDs. Of course, absence of evidence does not mean evidence of absence, but it confirms that REDs is a model that still needs to be thoroughly tested.

Since REDs is being diagnosed in sports practice, regular publications are appearing from major organizations like the IOC [3, 4, 48], and REDs clinics are being opened [6–8], the assumption is that the underlying evidence is strong. In this section we will make the point that this is not the case, and that especially concerning REDs, the ideas are so new, and the research required so complex, that many aspects of the model are only supported by associations, and findings are not consistent. In the evidence pyramid, clinical research trials provide the most control and can demonstrate causality. Studies that report correlations or associations cannot demonstrate causality. As we will see in the section below, very few studies have demonstrated causality between LEA and REDs symptoms. Most of the evidence describes associations that provide a much lower level of evidence, because the symptoms are not very specific, there are many different potential causes that would need to be considered in addition to LEA.

As discussed above, the error of assessment of EA can be very high, yet many cross-sectional studies are still based upon poorly measured outcomes that are likely inaccurate (and often an under-estimation of the real value). Much of the evidence purported to support the REDs model is therefore mostly obtained from indirect evidence, derived from studies that did not involve athletes, and often obtained with different research questions and objectives in mind. Arguably the strongest support for the model should come from the areas that have been researched the most, i.e., the parts of the female athlete triad: effects of LEA on reproductive function, energy metabolism, bone health and immune function. We will therefore focus on these areas in the following sections of this review to demonstrate that there are still many unanswered questions even in these relatively well studied areas.

### Reproductive Function

Here we critically discuss the available evidence regarding the relationship between LEA and reproductive function, including menstrual function, sex hormone dysregulation and sperm quality. While LEA can be a potent modulator of the HPG axis in men and women [30], other possible etiological factors should also be considered when an athlete displays signs and symptoms of reproductive dysfunction. Other causative factors may act independently of LEA or interact synergistically with LEA; consequently, focusing on EA alone may lead to other important causative factors being missed, hampering effective treatment. For example, psychological stress, depression, anxiety [54, 55], and poor sleep [34] often occur simultaneously with LEA [56] and have known influence on the hypothalamic–pituitary–adrenal (HPA) axis and may therefore modulate the HPG axis and reproductive function [57]. Accordingly, menstrual disturbances in women elicited by a diet and exercise programme were associated with metabolic stress but also with significant increases in perceived stress [58]. Elegant work, albeit in female cynomolgus monkeys and yet to be replicated in humans, showed much greater impairment in reproductive function (assessed as abnormal menstrual cycle length) after a combination of psychosocial stress plus diet and exercise stress compared with either stressor alone [59].

Animal models have historically provided strong support for a relationship between LEA and reproductive function. In female animals there is clear evidence of menstrual cycle suppression as a result of pharmacological inhibition of substrate utilization [60], increased thermoregulatory demand [61] and increased foraging effort [62] in rodents, and a high exercise volume without a compensatory increase in calorie intake in monkeys [63]. In male animals the evidence of alterations in reproductive function is less clear, but there are examples showing reduced sperm quality and disrupted testicular metabolism in rodents [64] and suppression of luteinizing hormone and testosterone pulse frequency in male rhesus monkeys [65]. However, there is also evidence of favorable effects of long-term calorie restriction improving testicular function in male rhesus monkeys [66].

In human females, the causal effect of LEA on sex hormone concentrations has been determined via systematic laboratory-based research emerging through the 1990 and 2000s [30, 67] ultimately putting LEA at the core of the female athlete triad model [2, 19]. Several lines of enquiry provide evidence of disruption to reproductive function due to LEA. Short term studies in exercising females provide direct evidence of disruption of the hypothalamic pituitary ovarian (HPO) axis hormones luteinizing hormone (pulsatility) and follicle stimulating hormone, but not oestrogen with LEA [30, 67]. In addition, to these mechanistic studies, a large body of observational human studies in female athletes [4, 28] associate disruption to reproductive function with LEA. Therefore, there is support for the idea that, in females exposed to LEA, the cyclicity of primary female sex hormones is blunted resulting in menstrual disturbances. Depending upon the severity and duration of LEA, amenorrhea may develop [59, 68], though the relationship between reduced energy availability and menstrual dysfunction in practice is less clear [37].

In human males*,* although direct supporting evidence is scarce, negative impact on male reproductive function may be evidenced through decreased testosterone, libido, sexual dysfunction and spermatogenesis and sperm abnormalities [19]. Testosterone is considered a key hormone in the development of reproductive dysfunction and secondary hypogonadism (clinical or subclinical), which are considered primary indicators of REDs [4, 69]. Although severe energy deficit in healthy individuals has been associated with reduced circulating testosterone [70] and sperm quality [71], it is not clear if exercise stress on its own may down-regulate circulating testosterone [69, 72] and what is the independent effect of LEA. In three LEA-specific experimental studies of 3–6 days duration in males, where exercise volume was not a confounding factor, LEA did not affect circulating testosterone [73–75].

For an athlete managing various training, competition and life stressors, LEA is likely to co-exist in the presence of a variety of other stressors. These other stressors may even be responsible for the disruptions in the HPA and HPG axis in the absence of/independent of LEA. Military studies lend themselves well to research in multi-stressor environments that could be applied to athlete settings. For example, women reporting high levels of psychological stress during intense military training, exhibited perturbations of the HPA axis [76], marked HPG axis suppression and prevalence of menstrual disorders without clear signs of LEA [77]. The authors are not aware of similar studies in female athletes highlighting factors other than LEA in the etiology of reproductive dysfunction.

In males it has been known for decades that psychological stress can reduce testosterone and spermatogenesis via central mechanisms [78]. Multi-stressor environments that include energy deficit reduce testosterone, that returns to normal levels when the stressors are removed [79–81]. Several studies of the longitudinal effects of training and competition and associated variables (sleep, the positivity of coaching, winning versus losing, competition environment) describe a modulatory effect on the hypothalamic pituitary adrenal (HPA) and HPG axis in male athletes (for example [82, 83]) that should be considered in future research and in applied practice.

In summary, variations in reproductive function are to be expected in athletes with numerous factors having an influence on the HPA and HPG axis in females and males. There is a need for well-controlled studies to determine to what extent, different stressors may contribute to reproductive health disturbances in athletes, and the point at which these may reach clinical concern. This research would help to establish preventative strategies and comprehensive clinical treatment. Further work is needed to establish the specific causal relationship between LEA and reproductive function in athletes.

### Energy Metabolism

Although LEA is often associated with “impaired energy metabolism,” it is not always clear what this represents. Within both the 2014 REDs consensus and 2018 update, the term “metabolic” is not specifically defined, but is stated as “impaired physiological functioning caused by relative energy deficiency and includes impairments of metabolic rate” [3, 28]. The 2023 REDs consensus update extends this to further include “exposure to problematic LEA, with detrimental outcomes including decreases in energy metabolism” [4]. From those studies used to evidence the evolution of the REDs models, it appears that “metabolic rate” and “energy metabolism” are describing perturbations that may occur at either the whole body and/or tissue levels. These can be assessed through measurements of resting metabolic rate (RMR_meas_) and other indirect markers, including the endocrine hormones of the hypothalamic-pituitary-thyroid axis (e.g., triiodothyronine; T_3_; see Table 2 of the 2023 consensus) [84].

There are important points to consider regarding these measures, as they are often used to indicate a “pathology” in relation with REDs. As an example, metabolic changes at the whole-body level, can occur independent of those at the tissue level via a phenomenon known as adaptive thermogenesis, defined as a reduction in RMR_meas_, beyond what could be predicted from changes in fat and FFM [85]. As this could demonstrate a compensatory response to LEA, it is a common practice in both research and applied settings to examine RMR_meas_ against an equation-based prediction of RMR (RMR_pred_), to establish a ratio (RMR_ratio_). If this ratio is below an arbitrary threshold (i.e., < 0.90) this is considered as a proxy surrogate of energy deficiency [86]. Given the complexity of RMR_meas_ standardization procedures and the numerous RMR_pred_ equations used across male and female populations, the combinations of these two factors may warrant lower or higher cut-offs for RMR_ratio_ [87]. Furthermore, for measurement of indirect markers such as T_3_, clinically relevant reference ranges from across globally diverse population are difficult to agree, nor have these been well established in athletes [88]. A deviation from the average might be expected in an athlete population and does not necessarily indicate pathology, therefore, do we have clear cut off values for what is considered normal? Do we know when we are dealing with an adaptation versus development of pathology?

Besides these considerations, it is important to note that the evidence quoted to support the notion that the manipulation of energy intake and/or exercise energy expenditure may affect energy metabolism is not derived from athletes. Studies in this area are mainly conducted in lean healthy, overweight/obese, sedentary or active males and females, but not specifically athletes [30, 84, 87]. Furthermore, within the three consensus statements [3, 4, 28], many of the studies included to evidence any negative perturbations on markers of energy metabolism include those that have not directly examined LEA per se [89–93] or were conducted in cohorts of exercising females with a focus on outcomes predominantly related to the female athlete triad and not specifically REDs [59, 94–98]. The evidence therefore is indirect.

In addition, the majority of studies are cross sectional or longitudinal making it impossible to establish causality of LEA. There are also many conflicting outcomes. Some studies have highlighted an apparent relationship between LEA and negative perturbations to markers of energy metabolism (i.e., reduced RMR_meas_, RMR_ratio_ and/or T_3_) [99–102], with others showing no effect, but with individuals not being in LEA (above the threshold for LEA) [103–105]. Other studies show no negative outcomes despite LEA [17, 43, 46, 106–108].

Experimental studies have highlighted a potential causal link between the effect of LEA on energy metabolism in recreationally trained eumenorrheic females [109], yet this was not shown in well trained male endurance athletes [73]. The time course of these studies was short (3–10 days) and any highlighted significant differences were small (65 kcal∙day^−1^ in RMR_meas_) and not below clinically relevant reference ranges (> 1.0 nmol∙L^−1^ T_3_). If we use these markers to indicate pathology, what evidence is there to confirm that this indicates pathology and not adaptation. In other words, what would be classified as adaptable versus problematic LEA in this instance?

In summary, energy balance and likely also energy availability are dynamic processes and maintenance of body mass is the result of a highly complex and dynamic energy balance system, in which perturbations to individual components can cause coordinated and inter-related compensatory responses elsewhere. The strength of these compensatory responses is individually subtle, and early identification of this variability may help recognize individuals that respond well or poorly to an intervention. Changes in RMR_meas_ may deviate from RMR_pred_ owing to a range of methodological reasons, and/or because RMR_meas_ is not static. We have little or no evidence to support that a deviation in an arbitrary cutoff (i.e., RMR_ratio_) or changes in indirect markers (i.e., T_3_) indicates a pathology. This in combination with the fact that even the existence of adaptive thermogenesis is still heavily debated [110], means that these measures are not a solid evidence-based indicator of pathology that can be used in the diagnosis of a syndrome. Further experimental research is necessary for thorough characterization of the relationship between of LEA and measures of energy metabolism, specifically in athletic populations. We would also encourage future research that investigates whether the changes that are observed are “normal physiological responses,” “adaptations,” or “pathology.”

### Bone Health

The bone health of athletes was the subject of interest and research even before the female athlete triad was proposed in 1993 [1]. In the male and female athlete triad and REDs models, LEA is linked to adverse bone health. Herein we will make the point that, whilst there is evidence of an effect of LEA on bone outcomes, this evidence might not be as strong as one might imagine, especially given the difficulties in:

1. measuring energy availability;
2. determining the bone health of athletes;
3. isolating the effects of LEA from other factors (i.e., exercise factors, nutrient availability, sleep, illness, psychological stress);
4. determining the extent to which short-term periods of LEA and adaptations to bone relate to longer-term problems for bone health.

Much of the evidence base in this area comes from clinical populations, particularly those with anorexia nervosa (AN), but also from those with other eating disorders (albeit to a lesser extent), where altered bone metabolism, low BMD and increased fracture risk have been reported (see [111–113] for relevant systematic reviews and meta-analyses). It is difficult, however, to extrapolate findings from non-athletic individuals with eating disorders to athletes experiencing LEA, even when the athlete is also suffering from an eating disorder. Differences in diet composition and nutrient intake are likely to exist (which could influence bone outcomes). A further difference is that the LEA state experienced by the athlete is underpinned by significant energy expenditure through exercise, which is different (type, intensity, duration, volume) to some, although certainly not all, individuals with an eating disorder [114]. Exercise could alter the effects of LEA on bone, although not necessarily consistently in the same direction depending upon the type of exercise performed (e.g., [115–117].

The case that LEA directly influences bone is mostly based upon studies that examine changes in bone markers in short term studies. These studies investigated bone markers either acutely or over short periods of LEA lasting between 3 and 5 days and showed that changes in bone marker concentrations can occur within this timeframe [32, 115, 118]. LEA tends to reduce circulating concentrations of bone formation markers (for a review see [119, 120]) and increased bone resorption marker concentrations might also occur with more severe reductions in energy availability [32].

Although short term studies tend to support an effect of LEA on bone, it is difficult to determine how important this might be for longer-term bone health (i.e., over months or years). It remains unclear whether the acute and/or short-term responsiveness of bone markers is consistent following the repeated application of the same stimulus over time. Most importantly, it is not clear whether changes in these markers can predict longer-term alterations to bone mass, microarchitecture or bone stress injury risk. Until these elements are confirmed, it is difficult to determine whether the changes that have been shown in bone marker concentrations are positive or negative for longer-term bone health and whether they are clinically relevant for the athlete. In the context of the definitions provided in the latest REDs consensus statement [4], these acute and/or short-term studies would seem to be more reflective of adaptable responses to LEA rather than of problematic responses to LEA.

As such, and somewhat understandably given the difficulties in conducting long-term studies in this area, the available evidence for an effect (or not) of LEA on bone outcomes in athletes largely comes from cross-sectional studies. Here, conclusions are drawn on the basis of various bone outcomes in athletes who are reported to be in LEA when compared with those who are not (for a selection of examples see [43, 101, 121–127]). These studies tend to show reduced BMD at some (but not all) sites and some (but not all) studies report increased bone stress injury risk with reports of LEA and/or associated indicators. Whilst cross-sectional studies can be useful in the generation of hypotheses and in informing further study designs, the major issue is that they cannot establish a cause-and-effect relationship or provide information on how outcomes and behaviors might change over time; they essentially just provide a snapshot of one point in time. A further issue is that, whilst these studies tend to use relatively strong measures of bone related outcomes (e.g., dual energy X-ray absorptiometry derived measurements of BMD, high-resolution peripheral quantitative computed tomography derived measurements of bone micro architecture and strength, bone stress injury records), they rely upon relatively weak indicators of LEA (e.g., diet and training records, Triad or REDs symptoms, LEA questionnaires).

A further important consideration when evaluating the effects of LEA on bone is the fact that it is often hard to separate the effects of energy availability from the effects of nutrient availability. In terms of athlete bone health, this is perhaps most apparent with regards to carbohydrate and calcium availability, but likely also relates to other nutrients of importance for bone (for a broad overview of these see [128]).

It is extremely difficult to identify a magnitude and duration of LEA that might predispose an athlete to bone problems. Determining the direct impact of LEA on athlete bone health that is intermittently applied or not continuously applied over the longer-term, is even harder. The current evidence for an effect of LEA on bone health is not based on optimal study methods or designs, which, for example, makes it difficult to separate the independent effects of LEA from numerous other factors. There is a need for further research that recruits larger numbers of athletes and utilizes gold standard methods to significantly improve our understanding. It is noted, however, that this would be a major undertaking and the feasibility of such studies is questionable.

### Immune Function

Evidence is currently lacking to support the notion that LEA of the magnitude often reported in athletes causes ‘immunological dysfunction’ [129, 130]. A handful of cross-sectional, survey studies show that leanness and LEA are associated with the recall of illness symptoms in athletes [16, 56, 131]. Besides the acknowledged challenges assessing energy availability in free-living athletes, and the lack of a clear definition for LEA [33], these studies suffer many of the limitations common to field investigations of athlete immune health, including:

1. absent or inadequate comparator control group;
2. confounding owing to recruitment bias and low responder rates;
3. unstandardized recall of illness symptoms over the last 1–3 months, rather than recommended daily symptom monitoring using validated questionnaires (*e.g.*, Jackson or Wisconsin) [132];
4. investigating (LEA as a risk factor for) only a small number of self-reported respiratory symptom episodes occurring outside of the common cold season (e.g., 16 respiratory symptom episodes among 81 athletes in the Australian summer [131]);
5. lacking pathology tests to confirm symptoms were of infectious etiology (e.g., rather than seasonal allergy) [133] and
6. lacking in-vivo immune measures that provide clinically relevant information about “immunological function” (e.g., vaccination responses) [132, 134].

Reporting only time lost from training due to illness symptoms in the last month does not constitute a rigorous and comprehensive immunological assessment sufficient to determine “immunological dysfunction,” a term used in one recent paper examining REDs symptoms in elite and pre-elite athletes [16]. Similarly, reporting only circulating leukocyte counts after laboratory exercise does not provide an adequate assessment of the “immune response” to exercise with LEA [75]; researchers are directed to the methodological recommendations of the Exercise Immunology Society [132, 134].

**Fig. 2:**
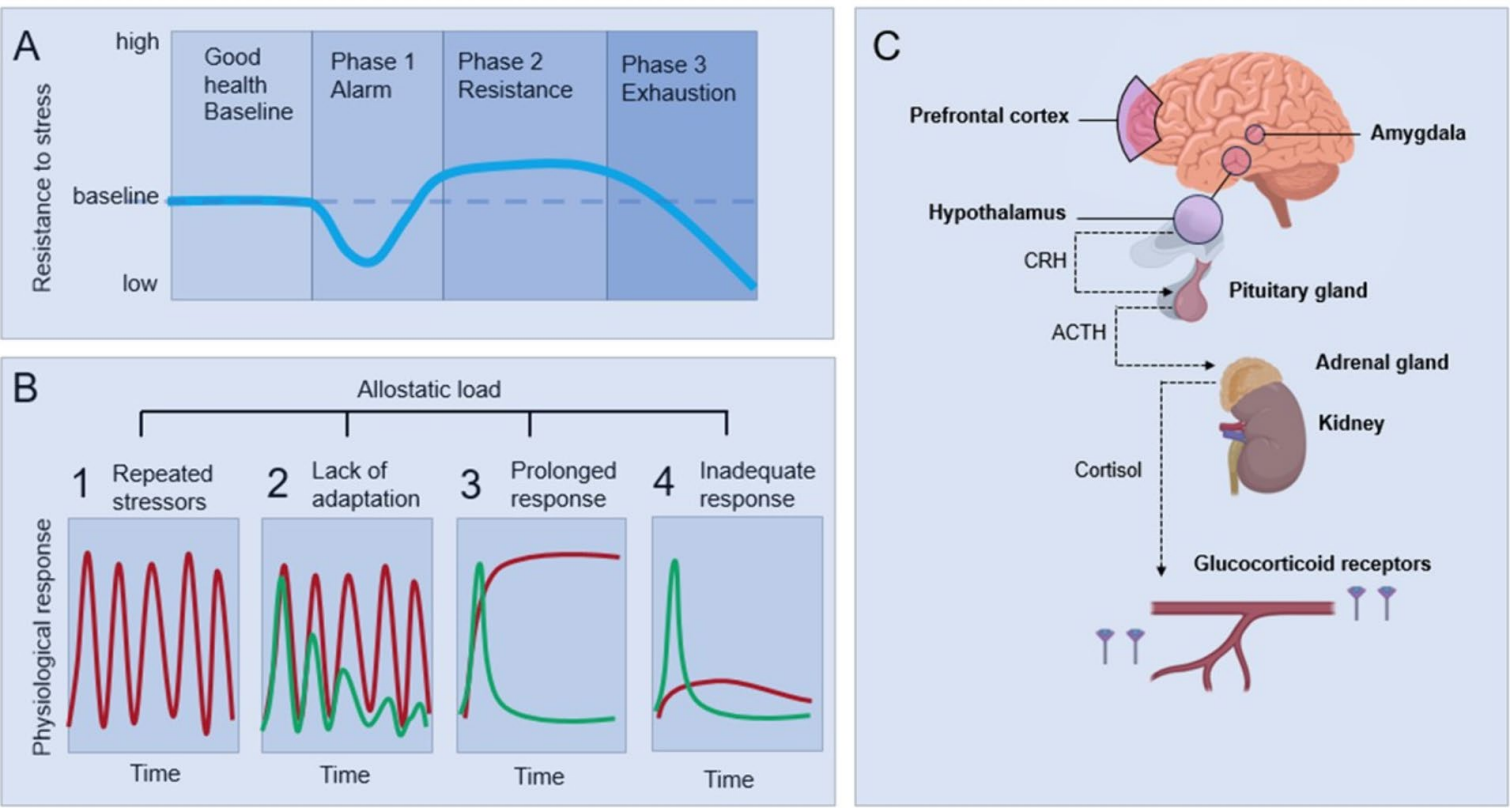
The general adaptation syndrome (GAS) (**A**), allostatic load model (**B**), and the pathway (**C**) that connects various stressors with symptoms and pathology. Effective coping to any stressful situation depends on the person’s cognitive appraisal of the stressful event (perceived stress), and the subsequent type of behavioral coping strategy used [167]. The GAS model (**A**) described how a stress response causes function (represented by the blue line) to decrease initially (phase 1). Adaptation occurs and this helps to resist the continued stress (phase 2). After a while this cannot be sustained anymore and exhaustion occurs (phase 3). The GAS model also explained the role of common neuroendocrine pathways for various challenges (sympathetic nervous systems and HPA axis) (**C**). The GAS model was further developed into the allostatic model (**B**) [169, 171]. Allostasis is a response to challenges whereby the setpoint will change. The most well studied allostatic responses to a range of challenges involve the sympathetic nervous systems and HPA axis (**C**). Activation of these systems, independent of the source of stress, releases catecholamines from nerve endings and adrenal medulla and leads to corticotropin secretion from the pituitary gland. Corticotropin, in turn, will stimulate the release of cortisol from the adrenal cortex and this will exert its effect through binding to glucocorticoid receptors in various target tissues, which can be up or downregulated. This is a fast and effective response. If this response is not immediately turned off, over time this increases the “allostatic load.” Allostatic load or eventually overload will affect many body systems causing wear and tear [169, 171]. Four situations are associated with allostatic load (panels B1–4; the red line represents problematic responses and the green line normal responses). The first and most obvious is frequently repeated stress (B1). A second situation would be inadequate adaptation to repeated stressors of the same type (B2). This can also result in prolonged exposure to stress hormones. The third would be a situation where there is an inability to shut off the stress response after termination of the stress (B3). An example of this is exercise training that induces allostatic load in the form of elevated sympathetic and HPA-axis activity, which may result in weight loss, amenorrhea, and even AN [182]. In the fourth type of allostatic load, there could be an inadequate response by one system and this could trigger a compensatory increase in another (B4). For example, if cortisol secretion fails to increase in response to stress, secretion of inflammatory cytokines (which are counter-regulated by cortisol) increases [183]. A range of challenges that an athlete faces (training load, competition stress, poor nutrition, poor sleep) can increase allostatic load, which can trigger a range of symptoms and clinical conditions

Prominent risk factors for infection in elite athletes and military personnel are similar to the general population and include wintertime (i.e., common cold and flu season) [135, 136] and foreign travel [136], when exposure to pathogens increases, alongside factors that influence neuro-endocrine-immune interactions including stress, anxiety and depression [131], high training load [137] and poor sleep [138, 139]. Psychological stress, poor sleep, and heavy exertion influence immune function via the shared effector limbs of the sympathetic-adrenal axis and pituitary-adrenal axis and the resulting immunoregulatory hormones, such as cortisol (Fig. 2) [140]. It stands to reason that the emotional experience evoked by different situations (i.e., overloaded versus coping, aversive versus pleasant) influences immunity and illness incidence in elite athletes during heavy training and major competition. Indeed, subjective stress and anxiety have been shown to modify the immune response to exercise [141]. Given the shared pathways for the body’s response to various psychological and physical challenges [142], it is not at all surprising that studies reporting greater recall of illness symptoms in female athletes with LEA (LEAF-Q score ≥ 8) show considerable interdependence of various illness risk factors [56, 131]. For example, anxiety, stress, depression and overall recovery state explained 74% of the variance in illness symptom episodes in one study [56] and the odds ratio (OR) for illness was stronger for depression (OR = 8.4) than LEA (OR = 7.4) in another [131].

Nutrient availability influences immunity because macro- and micronutrients are involved in a multitude of immune processes; macronutrients are involved in immune cell metabolism and protein synthesis (e.g., production of immunoglobulins, cytokines, and acute-phase proteins) and micronutrients in antioxidant defenses (for review see [129]). So long as the diet is made up of a diverse variety of foods, ensuring no nutrient deficiency, dietary energy restriction typically elicits a healthy phenotype reducing the incidence of diabetes, cancer and cardiovascular disease and extending the lifespan [143–145]. Studies on LEA in recreational and military populations show only subtle and short-lived changes in immune markers, and no changes in the immune-modulating hormone cortisol, after short-term severe energy restriction (48 h, ~ 90% restriction) [146, 147] and long-term moderate energy restriction during training (8 weeks, ~ 25% restriction) [148]. Although severe dietary carbohydrate restriction (< 10% dietary intake as carbohydrate) is associated with an exaggerated cortisol response to exercise [75, 149, 150], LEA appears to have a limited effect on circulating cortisol in athletes [151]. This finding is in keeping with a meta-analysis showing that circulating cortisol increases in states of complete fasting but not typically during less severe energy restriction [152].

Protein deficiency is widely considered responsible for the clinical features of immune suppression in severely advanced Kwashiokor and starvation [153], supported by protein-energy malnutrition research in animals [154] and the observation that immunity is typically well-preserved in patients with AN is likely because protein intake is adequate (carbohydrate and fat are typically reduced) [129, 155]. In female endurance athletes with an LEA determination [body mass index (BMI) 18.9 kg/m^2^] protein intake appears to be more than adequate to support immunity, typically exceeding government (0.8–0.9 g/kg/day) and endurance athlete specific recommendations (1.2–1.7 g/kg/day) [46, 156]. Paradoxically, AN is considered to afford protection against infection, that is until the condition becomes extremely severe (BMI < 15 kg/m^2^) when cross-sectional studies report evidence of decreased cellular immunity [157–159]. Moreover, hospitalized patients with AN suffer infections readily during rapid refeeding protocols [160, 161]. As food restriction and exercise are considered anxiolytic in AN, with as many as 83% of patients reporting a history of anxiety disorders [162], eliminating these anxiolytic behaviors in hospitalized patients with AN undergoing rapid refeeding likely accounts for the clinical levels of psychological stress, depression and anxiety, and decreased immunity [163]; indeed, psychological stress is widely accepted to increase susceptibility to infection [164].

In summary, direct evidence supporting the notion that LEA in female athletes causes “immunological dysfunction” is currently lacking. Athlete immune health should be considered in a broad conceptual framework that encompasses mental health (e.g., anxiety, stress and depression), sleep, recovery status, and nutrition [56, 142].

### Conclusion on the Strength of Evidence for the REDs Model

REDs was proposed in 2014 as a model to explain the effects of LEA; over the years it has expanded from being the cause of ten problem areas [3, 28] to now 14 categories of symptoms [4]. Of the many symptoms in the REDs model (Fig. 1), those discussed above are amongst the most researched. It is clear though, that even in these more researched areas, there are still many unanswered questions, in particular about the causal links between LEA and these symptoms or clinical manifestations. The evidence for the remaining symptoms (the symptoms not discussed here) is even less convincing, which is to be expected, given the fact that the model is nascent and performing longitudinal studies is time consuming, complex, and likely expensive. Perhaps this is also why in the latest consensus statement there is mention of theoretical, empirical and clinical evidence to support the model. We should focus on the clinical evidence, but this evidence is limited and, in some areas, not available. Nevertheless, diagnostic tools, based upon symptoms are being developed and used. A recent review listed as many as eleven different diagnostic tools to identify LEA or risk of REDs [53]. In the next section we will discuss alternative explanations for these symptoms as well as reasons why it is unlikely that a single factor can fully and independently explain the symptoms described as REDs.

## Alternative Explanations for REDs Symptoms in Athletes

As acknowledged in the recent REDs consensus statement, there are alternative explanations for the symptoms described by the REDs model [4]. The statement also mentions differential diagnoses for the first time and these are discussed in more detail in a supporting paper [52]. There are other causes of the same symptoms that need to be excluded before we can draw conclusions about the relative importance of LEA. It is highly likely that in almost all situations the causes are multifactorial, especially because the pathways for neuroendocrine responses to challenges an athlete faces (exercise load, competition, sleep disruption, travel, etc.) are shared via the HPA axis. This was first recognized in 1936 in a letter by Selye to the editor of Nature [165] and in 1950 when Selye [166] published a paper on the general adaptation syndrome (GAS) model (Fig. 2A). According to Lazarus and Folkman’s [167] model of stress and coping, effective coping to any stressful situation, depends upon the person's cognitive appraisal of the stressful event, and the subsequent type of behavioral coping strategy used. Sterling and Eyer built on the idea of the GAS model and introduced the paradigm “allostasis” in 1988 [168]. This was further developed by McEwen in subsequent years [169–172]. The allostatic model described how one, or many different stressors combined, can result in an increase in allostatic load. Over time this causes “wear and tear” on one or many systems in the body, emerging as a disease state if left unchecked. The parallels between the disease states in the allostasis literature and the multitude of REDs symptoms are apparent [169–172].

In sport science, everyone is familiar with the term homeostasis (originally described by Claude Bernard as “milieu interieur”) [173]. No one argues with the idea that maintaining homeostasis is critical to optimizing good health. Body temperature, serum glucose concentration, serum sodium concentration and blood pH are a few examples of variables that are controlled within a very narrow physiological range and if this control fails for some reason, there will be severe health (even fatal) consequences.

Allostasis is another type of process that is critical to survival [168]; but perhaps not so well known to most sport scientists and certainly largely ignored in the sport science literature. Allostasis means “achieving stability through change”. Allostasis is a response to a change from outside (or inside) the body, but a fundamental difference with homeostasis is that allostasis is not trying to restore parameters to their “setpoint,” instead, the setpoint will change. The most well studied allostatic responses involve the HPA axis (Fig. 2C). If there are many frequent challenges, if the stress response is not immediately turned off, if responsivity of the stress is affected or if alternative pathways are activated (Fig. 2B), this increases the “allostatic load” over time. Over weeks, months, or years, exposure to increased secretion of stress hormones, such as cortisol (from whatever source of stress), can result in allostatic load [174] and its pathophysiologic consequences (Fig. 2). The damage and pathology of various body systems is often described as “wear and tear” [169, 171]. For example, past or current depressive illness is associated with lower BMD in women likely because the allostatic load of chronic, moderately elevated serum cortisol concentrations inhibits bone formation [175].

**Fig. 3:**
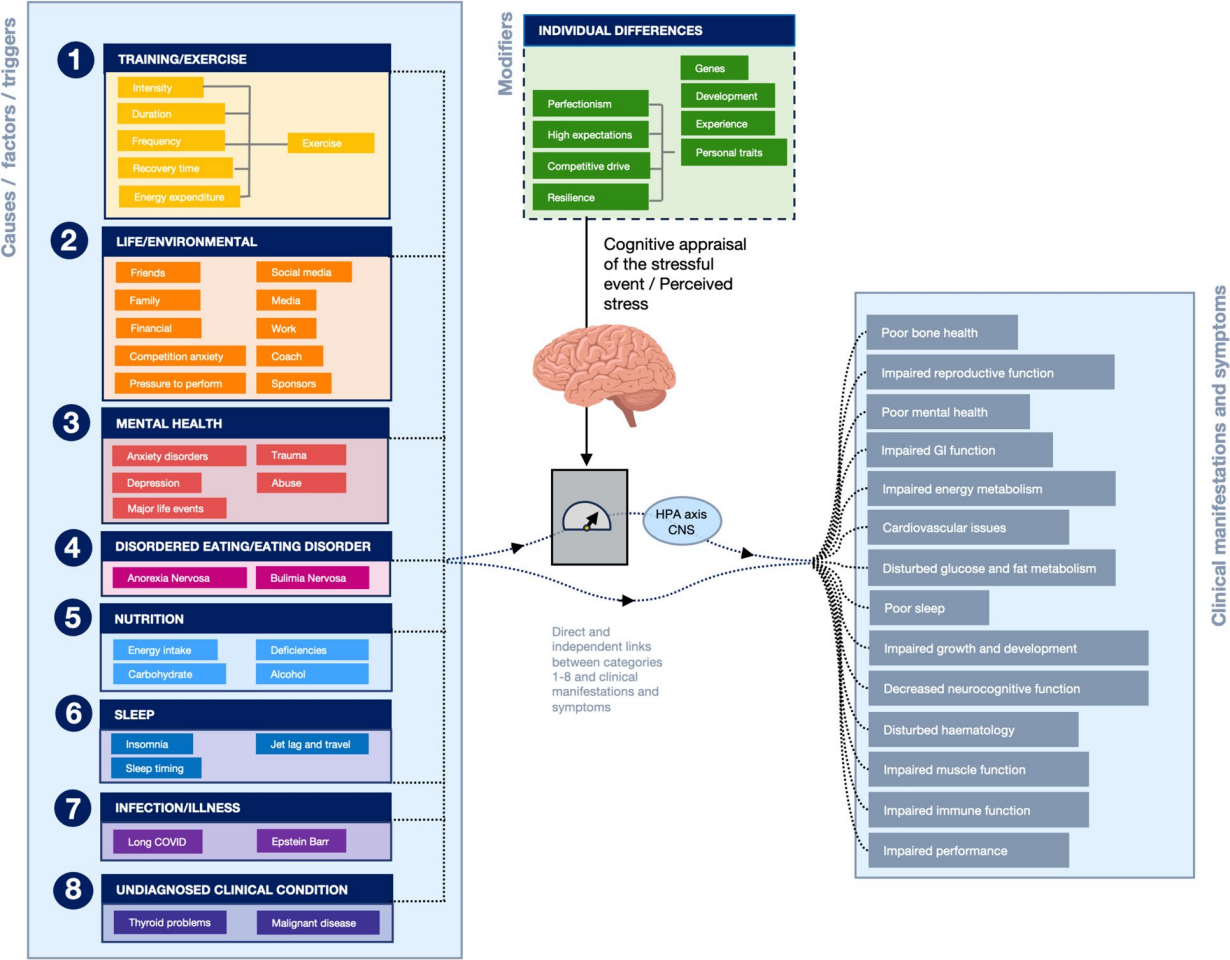
Common symptoms and clinical conditions in athletes that are similar to REDs may be caused by many factors independent of—or in combination with—LEA. Eight categories of factors that can contribute to these symptoms are shown (in no order preference). In many situations several factors, potentially from several categories, may play a role in the development of REDs symptoms in athletes. Many different types of challenges can independently or in combination increase allostatic load and over time this can cause wear and tear on the body and ultimately result in symptoms and pathology. The common pathways are the HPA axis and central nervous system (CNS). The brain plays a central role and psychiatric disorders, trauma, and abuse, as well as major life events, play an important role by modifying neuro-endocrine reactivity to stress. Life/environmental factors that can cause stress related to relationships, competition or self-image, to name just a few. There are also many important behavioral factors, most notably for athletes, including their training, their nutrition and sleep. Lingering infections can also affect allostatic load, but could also have direct effects on a number of symptoms. This is the case for several other factors as well; for example, iron deficiencies or other nutritional deficiencies can have direct effects, causing REDs symptoms

Because the mechanisms operate through the autonomic nervous system and the HPA axis that control most systems in the body, it is not surprising that most body systems are affected. An increase in allostatic load can affect the immune system, the gut, fatigue, and performance, reproductive function, bone health, sleep, neurocognitive performance, energy metabolism, glucose and fat metabolism, incontinence and more [169, 171] (see Fig. 3).

### Applying the Allostatic Load Model to Athletes

In the 1990s, work on the overtraining syndrome (OTS) was published, the majority as reviews [176–179]. Excessive training was linked to a wide range of symptoms [179], similar to the symptoms of REDs [3, 48]. Many of them are generic symptoms [180]. In other fields of research, such as “stress” and “sleep,” many similar links with similar clinical outcomes and symptoms are reported [8, 169–172, 181]. The overlap between REDs, overtraining, stress, and sleep has not gone unnoticed and Stellingwerff et al. [180] concluded that many of the previous training studies did not control for energy intake and perhaps the reported symptoms were the result of LEA and not overtraining [180]. This conclusion, however, would seem biased towards LEA being the cause of all symptoms and that all factors are just moderators of this effect. It could also be interpreted as, in these multi-stressor situations, the accumulated stress (i.e., allostatic load) causes pathology over time because of the wear and tear it produces. LEA could be one of these stressors. Perhaps in studies that mention LEA and REDs, the effects of very high training volumes, poor sleep, or other stressors were underestimated?

Very few studies have applied the allostatic model to exercise but in the general stress literature there are many studies that provide support for the various aspects of this model [171, 184–188]. Figure 3 conceptualizes how we apply the allostasis model to athletes. The figure demonstrates that multiple causes (on the left side of the figure), likely in combination, can affect many body systems and cause a range of symptoms. It also visualizes the many factors that can act independently or in combination to cause such symptoms with common pathways being the HPA-axis and CNS. In real life it will be virtually impossible to untangle the effects of LEA from a wide range of other causes with similar outcomes (symptoms).

Athletes are exposed to specific challenges (stressors related to their sport) as well as common stressors that anyone would experience. Examples are captured in Fig. 3 under the name environmental stress. Examples of specific challenges for an athlete would include: exercise (training) most days of the week, sometimes several times a day; manipulation of their body mass or composition through under-fueling or avoiding carbohydrate. Athletes may feel pressures from social media to look a certain way and this may cause body dissatisfaction and anxiety [45]. Athletes will often feel pressure to perform, have to manage relationships with their coach, fellow athletes and sponsors. Athletes may also train at altitude or in extremely hot conditions.

Many athletes are perfectionists, achievers, highly competitive and have high expectations. Studies have shown that an athlete’s personality characteristics (e.g., tendency for perfectionism, high trait anxiety) influence their response to cumulative life stressors, predicting long-term negative health outcomes [189]. Chronic stress is associated with exaggerated neuroendocrine responses to acute psychological stressors (Fig. 3) [190] and recent life stress influences the immune response to exercise [141]. Another important consideration is early life adversity (ELA), which is more common in elite athletes than the general population and even considered a precursor for success in super-elite athletes [191, 192]. Individuals with ELA experience exaggerated emotional and neuroendocrine-immune reactivity to daily life challenges and are more susceptible to the long-term wear and tear associated with high allostatic load [193, 194]. The signature left by ELA increases vulnerability to immune dysregulation, inflammation, and long-term health consequences in adulthood; including, depression [195], generalized anxiety disorder [196], eating disorders [197], and poor sleep [198].

Athletes who are characterized by a combination of high athletic identity, perfectionistic concerns and negative life stress and poor coach-athlete relationship, were shown to be significantly more often affected by overuse injuries (74% of the time) [199]. It was noted that psychological factors may contribute to the risk of overuse injuries through complex interactions rather than through their independent influences [200]. In addition to specific stressors, athletes are also exposed to common life stressors that everyone else is exposed to, like relationship issues, financial or family issues, a lingering infection, an injury, tooth problems or having an unbalanced diet with nutrient deficiencies.

Individuals cope with various challenges in different ways (partly as a result of resilience) and it is the perceived stress in particular that appears to be a deciding factor in determining the allostatic load and potential consequences [201]. To some degree how someone deals with these stressors may be genetic, but experience and personal development can also help to modify the perceived stress [169, 170]. All stressors will activate the autonomic nervous system and/or the HPA axis and can influence metabolic, immune and cardiovascular responses. Each of these factors individually may not be very significant or impactful, but together these stressors can have synergistic effects that will add up over time (weeks, months or years) to increase allostatic load [171, 172, 202]. It is impossible, and from a practical point of view a futile exercise, to isolate a single stressor, as it is the accumulated stressors that determine the allostatic load. Please note that energy intake is listed under nutrition and energy expenditure under exercise as possible stressors; together these makeup energy availability. Exercise can cause a range of physiological responses (increases in stress hormones, cytokines, inflammation, activation of the HPA axis, increased core temperature, and so on), which may contribute positively or negatively to allostatic load. Exercise is not just a form of energy expenditure. Indeed, allostasis also comes with an energetic cost [203].

### AHaRC (Athlete Health and Readiness Checklist)

The listed symptoms of REDs are generic and can have many different causes. The symptoms described as “REDs symptoms” could also be described as symptoms that are common in sport and even outside of sport, not all of them and possibly few are caused by LEA. The new IOC consensus statement recognizes the fact that there could be other causes of the symptoms too, stating: “each of these outcomes can occur in the absence of LEA, therefore the differential diagnosis should be considered in the assessment and diagnosis of REDs severity and/or risk” [4]. Therein, an extensive list of such differential diagnoses is provided alongside a long list of common generic/common symptoms that athletes may display. The list, however, is perhaps too large for practical purposes and does not even cover all possible diagnoses, the tools for these differential diagnoses are not always clear (for example, how do you diagnose overtraining syndrome?) and the suggested approach [4] does not acknowledge allostatic overload. And most importantly the starting point of the proposed approach [4] is biased: “it is LEA, unless it is not.”

**Fig. 4:**
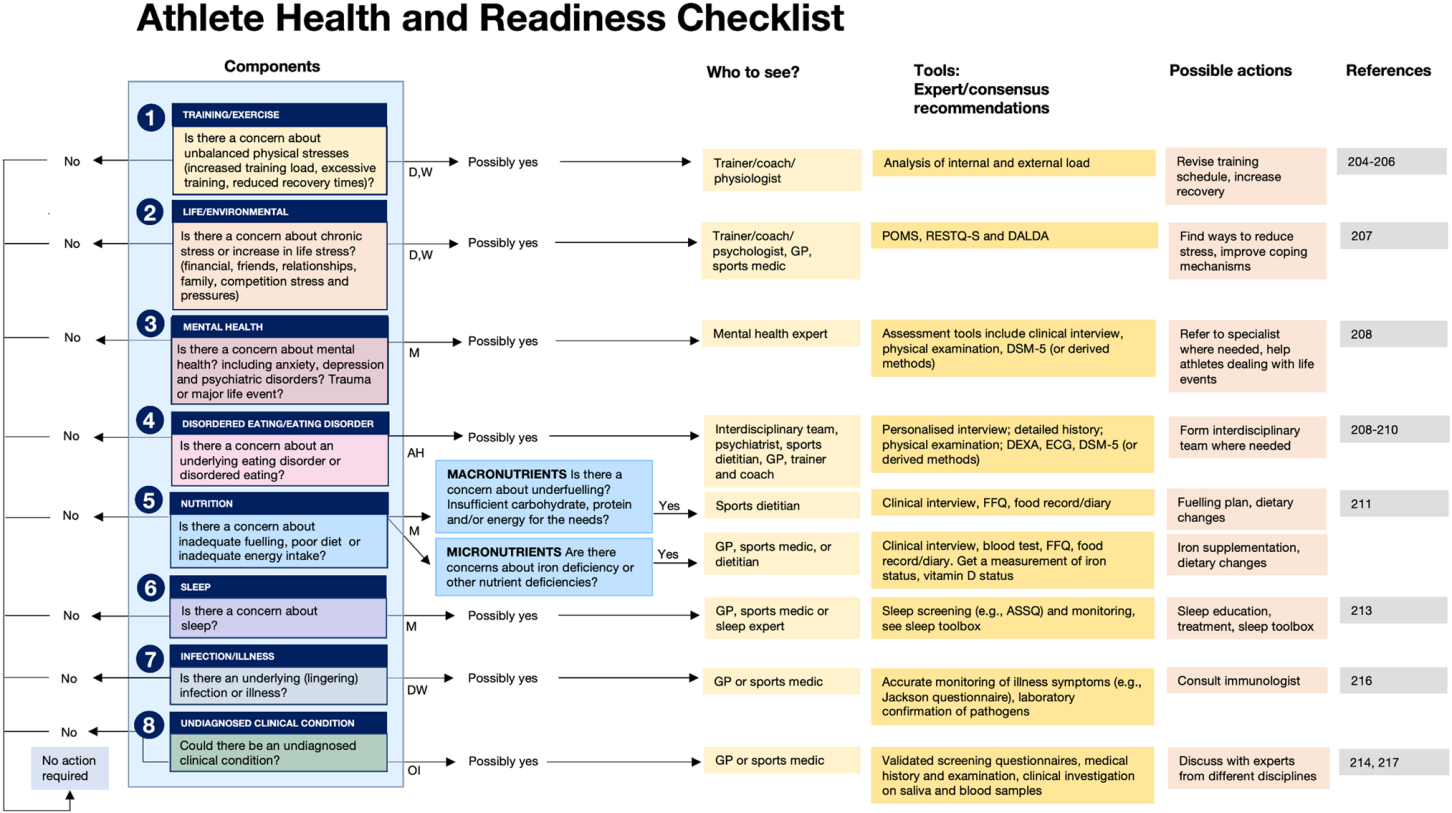
Athlete Health and Readiness Checklist (AHaRC) providing a multidimensional decision tree to maintain athletes’ health and performance. The AHaRC will act as a guide for practitioners working with athletes, to implement regular checks, identifying possible tools and the most relevant professionals to consult. There are eight categories (no order of preference), all important to check. Some need frequent checks (daily or weekly) others more periodically (suggested frequency: D = daily, W = weekly, M = monthly, AH = ad hoc, OI = on indication). The list here is not exhaustive but should be a good starting point for those responsible for athlete health. For each component in the checklist, the recommended tools and possible actions are supported by expert/consensus recommendations. Profile of mood states (POMS); recovery stress questionnaire for athletes (RESTQ-S), and daily analyses of life demands of athletes (DALDA). (1) Training/exercise [204–206], (2) life/environmental [207], (3) mental health [208], (4) disordered eating/eating disorders [208, 210], (5) nutrition [211], (6) sleep [213], (7) infection/illness [216], and (8) undiagnosed clinical condition [214, 217]

Herein we would like to broaden the viewpoint to better support athlete health. We identified eight categories of factors that could alone or in combination with each other cause a host of clinical manifestations and symptoms that overlap greatly with those described as REDs symptoms. In the search for the most important cause(s) and appropriate treatment, it is important to consider all eight of these categories; although we recognize that some aspects will be more practical and easier to check than others and are, therefore, more likely to be checked regularly. Categories are not independent and many links between them exist. In most cases it may not be possible to identify a single cause but a checklist will help to identify all areas that could help towards treating the causes of the symptoms or prevent additional clinical issues (Fig. 4).

1. *Training/exercise:* training-related stress (e.g., training load, training intensity, monotony etc.) [204–206].
2. *Life/environmental:* non-training related stress (e.g., family stress, competition stress, stressors related to performance goals and expectations, relationship with a coach, sponsors, travel, time zones, climates, media, social media, body image, societal pressure, etc.) [207]. These stressors can add up and accumulate over time and increase allostatic load.
3. *Mental health:* this includes psychiatric conditions such as depression and anxiety disorders [208]. This also includes recent or current life stress, major life events and early life adversity. Mental health has been recognized as a cause and consequence of LEA [209].
4. *Disordered eating and eating disorders:* although there is obvious overlap with mental health issues above (e.g., anorexia nervosa is a psychiatric condition) we list it here separately also because disordered eating which may not be diagnosed as a clinical disorder, may still have long lasting consequences and usually precedes an eating disorder [208, 210]. Disordered eating may result in nutrition deficits which are listed separately under point 5.
5. *Nutrition:* low energy intake is one of these possible deficits, but carbohydrate availability and adequate protein intake are important as well; micronutrient deficiencies (e.g., iron, zinc, magnesium, calcium, B vitamins, and vitamin D) could also play a role [211].
6. *Sleep:* poor sleep and clinical sleep disorders may cause a wide range of symptoms. Long-term consequences of sleep disruption in otherwise healthy individuals include (but not limited to) hypertension, dyslipidemia, cardiovascular disease, weight-related issues, metabolic syndrome, type 2 diabetes mellitus, reproductive function, depression, and anxiety and immune dysfunction [212, 213].
7. *Infectious etiology:* ongoing fatigue and recurrent illness symptoms may have an infectious etiology (e.g., related to herpes viruses, Epstein Barr, long covid etc. [214–216].
8. *Undiagnosed clinical conditions:* including, for example, thyroid problems, diabetes, cardiac problems, and malignant disease [214, 215, 217].

### Does REDs Differ from Disordered Eating?

It is often stated that the symptoms listed under REDs can occur with or without a clinically diagnosed eating disorder. But can it also occur without disordered eating? Papers regularly refer to accidental, unintentional, or inadvertent LEA [4, 24]. In such cases athletes are not aware that they are not consuming enough energy for their activity levels. However, what are the chances that this “accidental” energy deficit is chronic and not just in periods where they may train harder, are too busy etc.? We have not been able to find any evidence that connects problematic LEA with accidental undereating. This would imply that hunger feelings must not be adequately regulated.

In any case, eating disorders (ED) [218] or disordered eating (DE) [219] are a highly prevalent problem in this population at risk for LEA, making it difficult to untangle the physiological effects of LEA from the psycho-physiological effects of DE/ED in cross-sectional studies. For example, a cross-sectional study evaluating risk of eating disorders in conjunction with field-assessed energy availability in 121 collegiate athletes reported that 81% (*n* = 98) had “LEA” (a prevalence likely inflated by the methodological issues highlighted earlier), and 76% (*n* = 92) displayed LEA with an eating disorder risk [220]. Another study evaluating long-term markers of LEA and DE/ED in 464 adolescent (13–18 years) female athletes, reported that only 6.5% (*n* = 30) showed markers of chronic LEA (low BMD), and 24 of these (5.2% of total) did not meet the criteria for ED/DE [221]. Importantly, of the 426 individuals assessed 40% reported with desire to lose weight (7%), disordered eating (31%), or an eating disorder (2%) [221]. In summary, current evidence is limited to support the idea that inadvertent under-fuelling is rife, or that problematic (and thus chronic) LEA can occur in the absence of disordered eating.

### Practical Aspects

We have identified several reasons why it would be premature to use REDs as a diagnosis or even as a model. Many parts remain relatively untested, there is no way to distinguish adaptable and problematic LEA apart from waiting for the symptoms to develop. The model in general is too calorie-centric and the name and definition of the model only acknowledge one cause. Models are by definition a simplification of reality. But when is a model oversimplified and less useful, and when is it simple, not entirely correct, but useful? In line with a famous quote from British statistician George Box: “all models are wrong, some are useful,” one could argue, that even if the REDs model is an oversimplification, it could still be useful. Perhaps the greatest value of the REDs model is that it is extremely simple, and it may help athletes understand that obsession with body mass and composition and continued LEA may be unhealthy. The REDs model may, therefore, seem a useful education tool. However, it remains an oversimplification and athletes should be educated equally on the other aspects of health and performance (e.g., related to sleep, recovery, mental health). The question is therefore: Is it a useful education tool that will help athletes to be less obsessed with body mass and eat more according to recommendations? We are not aware of evidence that would support this notion, but this is certainly a research question that needs attention, i.e., Does REDs education using the model help to prevent symptoms of REDs?

The counterargument would be that the model is less helpful, it will over-diagnose individuals and it will distract from the complexities of the multifactorial etiology (Fig. 3). The 2019 IOC consensus on Athlete’s mental health states that reframing an eating disorder and calling it something else (e.g., REDs) may reduce stigma [208]. A major risk is that a diagnosis of REDs might prevent appropriate treatment for some individuals. If athletes are diagnosed with REDs, often by minimally qualified individuals, they may therefore not accept the treatment that is required (e.g., seeing a clinical psychiatrist in the case of an athlete with an eating disorder).

Tackling LEA could help in many cases but assuming that symptoms are always caused by an inadequate energy intake for the amount of exercise, may close the door to, or at least distract from exploring other reasons why these same symptoms may develop. In the analogy we used previously where overfeeding sugar in combination with a positive energy balance will result in weight gain, tackling just sugar intake may or may not have a positive outcome. An approach that explores all potential causes and gives them equal thought, without a bias towards sugar, would likely result in more effective treatment. Besides this, in athletes who are supported well by qualified practitioners, they will already receive the advice to fuel appropriately.

If LEA is the single cause of REDs symptoms, treatment should be easy (increase energy intake, decrease energy expenditure). In reality, however, there are many underlying dietary and psychological factors that determine eating behavior, and these are in most cases the main etiology, not just “calories” as the definition of LEA dictates.

## Overall Conclusion

REDs is a relatively new model and many aspects of the model have not been thoroughly tested. The title of this critical analysis is “Does Relative Energy Deficiency in Sport (REDs) Syndrome exist?” We may never get to the answer to the question does REDs exist. The main limitation of the REDs model is in the name and definition. The REDs model, as it is often presented, places energy availability at the center and it is becoming more and more obvious that this is an oversimplification of causation of a very complex and multifaceted symptomatology. One may argue that a differential diagnosis should be performed to confirm that calories are the cause. But by doing this, a bias is already present. We would welcome a discussion about a change from REDs to a broader more holistic athlete health approach and we encourage the scientific community to not hold on to terminology at all cost, but be open minded and adapt to make the model more accurate and more useful.

The research in this area is complex and everyone who has performed studies in this area should be commended for attempting to untangle some of these complexities. We encourage research in this area, to gain a better understanding of the etiology of the symptoms that athletes present.

Future studies will need to demonstrate causal links between LEA and all symptoms of REDs, they must be large scale, carefully controlled studies, longitudinal in nature (month and years, not days or weeks) with all important variables like nutrition intake and training load, energy expenditure, but also all other potential stressors in an athlete’s life, illnesses, infections etc. carefully monitored. These studies would need to be performed in free living athletes where numerous aspects of their lives need to be controlled whilst maintaining ecological validity. To study causality EA would need to be manipulated. However, studying “problematic” LEA may not only be time consuming, complex, and costly, but also unethical. We will encourage funding bodies to support such work, but at the same time we hope that time and effort will be spent on developing pragmatic approaches that will help practitioners in the field to consider all aspects of mental and physical health of an athlete, as well as performance.

Regardless, there is an urgent question from the field, where athletes suffer from various symptoms (similar to those presented by the REDs model). The causes of these symptoms are unknown, but could also be nutrition related. We propose a more holistic approach and a checklist (AHaRC) that assesses eight areas. In each of these eight areas we make use of tools that have been proposed in previous consensus statements or expert opinion pieces. The factors in these eight areas could all result in symptoms that could be mistaken for symptoms of LEA (REDs symptoms). As mentioned before, the new consensus statement is in agreement with this but we argue that REDs tools are primarily trying to prove that it is LEA instead of trying to find potential causes of the symptoms. It is, therefore, important to check all potential causes and have individuals with relevant expertise and qualifications to assess or triage the various components. As part of this larger assessment, nutrition and exercise/training patterns should be considered as a contributing cause, and as part of nutrition and exercise effects, energy or LEA should be considered. We are not dismissing the importance of energy availability at all; however, rather than having a calorie-centric approach where “LEA is the cause,” we are advocates of a more holistic, less biased approach that considers LEA as one of many possible causes, recognizing that it is also possible that accumulation of effects of various potential causes can result in negative side effects. From a practical point of view there is little or no need to identify LEA as a single cause. It is much more important to identify the origin of the presented symptoms.
